# BRAINSTORMING: A study protocol for a randomised double-blind clinical trial to assess the impact of concurrent brain stimulation (tDCS) and working memory training on cognitive performance in Acquired Brain Injury (ABI)

**DOI:** 10.1186/s40359-020-00454-w

**Published:** 2020-11-26

**Authors:** Sara Assecondi, Rong Hu, Gail Eskes, Michelle Read, Chris Griffiths, Kim Shapiro

**Affiliations:** 1 Visual Experience Laboratory, School of Psychology, University of Birmingham, Birmingham, UK; 2 Center for Human Brian Health (CHBH), University of Birmingham, Birmingham, UK; 3 Department of Neurology, Guangzhou First People’s Hospital, School of Medicine, South China University of Technology, Guangzhou, Guangdong China; 4 Departments of Psychiatry and Psychology & Neuroscience, Dalhousie University, Halifax, NS Canada; 5 Northamptonshire Healthcare NHS Foundation Trust, Northampton, UK

**Keywords:** Brain injury, Non-invasive brain stimulation, Direct current, Working memory training, N-back, Multi-session

## Abstract

**Background:**

Acquired Brain Injury (ABI) admissions have an incidence of 385 per 100,000 of the population in the UK, and as brain injury often involves the frontal networks, cognitive domains affected are likely to be executive control, working memory, and problem-solving deficits, resulting in difficulty with everyday activities. The above observations make working memory, and related constructs such as attention and executive functioning attractive targets for neurorehabilitation. We propose a combined home-based rehabilitation protocol involving the concurrent administration of a working memory training program (adaptive N-back task) with non-invasive transcranial direct current stimulation (tDCS) of the right dorsolateral prefrontal cortex to promote long-lasting modification of brain areas underlying working memory function.

**Method:**

Patients with a working memory deficit will be recruited and assigned to two age-matched groups receiving working memory training for 2 weeks: an active group, receiving tDCS (2 mA for 20 min), and a control group, receiving sham stimulation. After the end of the first 2 weeks, both groups will continue the working memory training for three more weeks. Outcome measures will be recorded at timepoints throughout the intervention, including baseline, after the 2 weeks of stimulation, at the end of the working memory training regimen and 1 month after the completion of the training.

**Discussion:**

The aim of the study is to assess if non-invasive tDCS stimulation has an impact on performance and benefits of a working memory training regimen. Specifically, we will examine the impact of brain stimulation on training gains, if changes in gains would last, and whether changes in training performance transfer to other cognitive domains. Furthermore, we will explore whether training improvements impact on everyday life activities and how the home-based training regimen is received by participants, with the view to develop an effective home healthcare tool that could enhance working memory and daily functioning.

**Trial registration:**

This study was registered with clinicaltrials.gov: NCT04010149 on July 8, 2019.

## Background

Working memory, which involves temporary maintenance and working with information in mind, is required for cognitive skills such as problem solving, planning and auditory and reading comprehension. Working memory operates across multiple modalities (e.g., auditory and visual), and is a system that combines attentional control with temporary storage and information manipulation [1]. A core concept of Baddeley’s [2] model of working memory is that it consists of multiple components: namely, a phonological loop governing for temporary maintenance and processing of verbal information, a visuospatial sketchpad to maintain and process visual and spatial information, an episodic buffer that bridges working memory and long-term memory, and a central executive that exercises attentional control over the above-named subsystems. A network involving the dorsolateral prefrontal cortex (DLPFC) plays a fundamental role in supporting working memory [3], with spatial tasks primarily involving the right DLPFC and verbal task involving the left DLPFC [4, 5].

Working memory is important for everyday tasks such as problem solving, reasoning, and learning [1] ; a deficit in working memory can lead to difficulties with many everyday activities that are necessary for work, study and general functioning [6]. Impaired working memory may consequently have a significant impact on quality of life and ability to participate in social roles, with potential for negative effects on mood and emotional wellbeing. Working memory is known to decline with aging [7] and is frequently compromised following brain injury [8].

Acquired Brain Injury (ABI) is brain damage caused by either a traumatic (e.g. a penetrating head injury due to an accident) or a non-traumatic injury (e.g., stroke, brain tumours, ischemia). ABI may be associated not only with physical but also with cognitive, emotional and behavioural impairments. Our research focuses on cognitive impairment resulting from ABI. As brain injury often involves frontal systems, cognitive domains commonly affected include: attention, executive control, working memory, and problem solving [9, 10]. Working memory impairment is highly prevalent across ABI aetiologies [11, 12] and severity levels. A recent meta-analysis of working memory deficits post traumatic brain injury revealed significant deficits in verbal short-term memory and visuospatial and verbal working memory (effect sizes ranging from .37 to .69 [11];).

The above observations make working memory, and overlapping constructs such as attention and executive functioning, important targets for neurorehabilitation, given the impact of these deficits on everyday function [6, 13]. Admissions for ABI have an incidence of 385 per 100,000 in the UK (https://www.headway.org.uk/about-brain-injury/further-information/statistics/). Individuals post ABI commonly report working memory dysfunctions [14, 15], thus solutions to improve recovery of working memory are critically needed.

Researchers have been exploring whether it is possible to train or improve working memory in clinical groups such as stroke patients [16], people with Parkinson’s disease [17], and children with attention deficit hyperactivity disorder (ADHD) [18]. For training to be considered of value, the gains in performance on the training task must transfer to other cognitive tasks or yield some benefit in daily function outside of the laboratory [19]. However, evidence for transfer to other cognitive tasks or daily activities is not yet consistently obtained [20].

Rehabilitation for working memory impairments can focus on compensatory strategies, which aim to compensate for the impaired functions by using those which remain intact, or supplementary external aids [21]. In contrast, recent studies have focused on increasing working memory capacity through extensive practice and training [19, 22]. Computerized working memory training has shown promising results in healthy participants [23, 24] as well as in ABI patients [7, 16, 25–27]. Mood measures also appear to improve following working memory training [21, 25]. One of the tools that has been assessed for its working memory training potential is the n-back task, which involves determining whether a new item in a sequence is the same as the one that appeared ‘n’ times before. While results have been mixed (likely due in part to methodological inconsistencies between studies [28], a dual n-back implementation wherein participants keep track of two streams of information (one audio and one visual), was successful in healthy participants [23, 24].

Brain plasticity, which can vary greatly by individual, plays a central role in the success of cognitive training. Transcranial direct current stimulation (tDCS) [29–32] is a non-invasive technique that is thought to modify cortical excitability which, in turn, can directly affect brain plasticity by making relevant brain networks more or less likely to fire synchronously. Synchronous firing has been suggested to modulate long-term potentiation (LTP) like plasticity at the synaptic level, a critical component for learning [33, 34].

The combination of cognitive training protocols and non-invasive tDCS has gained attention as a means for cognitive enhancement in both adults and older adults [35–37]. In young and healthy participants, anodal tDCS over the left or right dorsolateral pre-frontal cortex (DLPFC) improved working memory when administered concurrently with cognitive training using a n-back task, also showing transfer to other domains [38], as well as maintenance over time [39]. Preliminary evidence shows that the congruency between the side of stimulation, and therefore the functional specificity of the DLPFC, and the behavioural task plays an important role in training outcome [40, 41]. As many cognitive functions show a physiological decline even in healthy aging, combined tDCS/cognitive training protocols are of particular interest for older adults [37, 42, 43] but in the elderly results are mixed with some studies revealing null findings [35, 44, 45].

Despite encouraging results obtained in healthy populations, research involving brain stimulation in ABI patients is sparse. By stimulating over the left DLPFC, Kang et al. [46] obtained positive results on attention, albeit in a small sample. In a within-subject design, working memory performance in stroke patients can improve after anodal stimulation of the left DLPFC [47]. Villamar et al. [48] reviewed the potential of the therapeutic use of brain stimulation to modulate neuroplasticity in ABI patients; however, given the diversity of the nature of the lesions, more research is necessary to understand how to use brain stimulation effectively in ABI populations.

## Aims and objectives

The aim of this research project is to investigate the impact of combined working memory training and tDCS protocols in individuals following a brain injury in terms of the training gains, change in objective measures of working memory, mood and fatigue, as well as participants’ perceptions of day-to-day memory function and subjective benefit of the training program.

Specifically, our primary objective is to determine if the combination of tDCS and working memory training is superior to working memory training alone to improve working memory function in an n-back task in an ABI population. The key secondary objectives are to determine if the combination of brain stimulation with working memory training can:

- boost performance in other cognitive domains (attention, executive functions)
- speed training gains
- lengthen improvement duration to 1 month
- increase improvement magnitude

To address the above questions, we will use a randomized clinical trial, assessor- and patient-blinded with two parallel groups and a simple randomization with a 1:1 allocation ratio.

## Methods/design

### Study setting

Patients will be recruited from the Northamptonshire Healthcare NHS Foundation Trust (NHFT) by the Community Brain Injury Service, a multidisciplinary service that provides specialist neurorehabilitation to adults who have experienced an acquired brain injury. People accessing the service from which the participants will be recruited are aged 16 and over; the most common age group from an audit of last year’s referrals was 50–59, with the majority falling between the ages of 30–69. Aetiology of brain injury varies although traumatic brain injury accounted for over half of referrals last year, followed by stroke, tumours and then hypoxia and infection related ABI. The ratio of males to females was 60:40.

Training sessions with brain stimulation will be administered at home by a member of the research team (assessor), who will visit the patient at an agreed time for the first 2 weeks. During the remaining 3 weeks, patients will complete the cognitive training alone from home, via an internet-based application, while receiving a motivational catch up call every week. Weekends will be exempt from training. This home-based approach will help us reduce drop-out. Moreover, it will also increase convenience for the patient, many of whom may live some distance from the brain injury team base.

### Eligibility criteria

The study will recruit patients who experienced an ABI and have been referred to the Community Brian Injury Service of NHFT. Patients provide written, informed consent before any study procedure occurs (see Fig. 2) and must meet eligibility criteria (Table 1) at randomization. Assessors, who will also administer the brain stimulation, will receive training in the use of tDCS and basic life support.

**Table 1:** Eligibility criteria

**Inclusion Criteria**	
1. Referred to the service 2. Are between 18 and 69 years of age, inclusive 3. Have capacity and able to provide informed consent 4. Normal or corrected-to-normal vision and hearing 5. Having a working memory impairment (see screening procedure) 6. At least 3 months between the injury and the starting of the study 7. Has a computer or has access to a computer	
**Exclusion criteria**	
1. Pre-injury psychiatric or neurological disease by self-report (e.g., anxiety disorder, ADHD, Parkinson’s disease, etc.) 2. History of diagnosed severe depression (diagnosed pre-injury) 3. History of epilepsy (diagnosed pre-injury) 4. Family history of epilepsy 5. Have had fainting spells or syncope in the last 3 years pre-injury 6. Have significant hearing loss, vision or motor impairment that would prevent them from performing the task 7. Known to be pregnant 8. Consuming medication affecting cortical excitability or recreational drugs^a^ 9. Metal (except titanium) or electronic implants in the brain /skull (e.g., splinters, fragments, clips, cochlear implant, deep brain stimulation, medication pump…) 10. Metal (except titanium) or any electronic device at other sites in your body, such as cardiac pacemaker or traumatic metallic residual fragments 11. Have skin problems such as dermatitis, psoriasis or eczema under the stimulation sites 12. Have had brain stimulation in the past 6 months 13. Have undergone transcranial electric or magnetic stimulation in the past (more than 6 months) which resulted in adverse effects 14. Skull fractures, significant skull defects, skull plates or large vessels occlusions, no significant cortical lesion or atrophy at the site of electrode 15. Having had a seizure at the time of accident or between the injury and starting of the therapy.	

^a^We follow recommendations by McLaren et al. [49], and only exclude drugs that are shown to block tDCS effects (e.g., sodium channel blockers, calcium channel blockers, and NMDA receptor antagonist), while other medications shown to only modulate tDCS effects will be tracked and considered as covariate in the analysis. We will monitor for medication changes as a potential bias on the outcomes of the intervention and report any changes that may occur. If a patient become in need of medications shown to block tDCS effects, they will be excluded from further participation. We will monitor changes in medication and general health through in-person briefing during the first 2 weeks of the intervention, when a researcher is visiting the patient’s house, and via phone calls (e.g. “Have there been any changes in your health or medications since our last phone call?”) during the remaining 3 weeks of the intervention

### Recruitment

Forty adults between the age of 18 and 69 years will be recruited through the Community Brain Injury Service. Patients will be randomly assigned to one of two experimental groups: an active (or right-sided tDCS) group and a control (or SHAM) group. All patients will receive WM training. As there is evidence that age modulates individual responses to both tDCS [50–52] and training regimens [53, 54], the two groups will be age-matched in two age subgroups: those aged between 18 and 45 years and those aged between 46 and 69 years, randomised within age range. Other factors may also influence the impact of the intervention on patients, including medications, time since injury, baseline performance, and these factors will be included as regressors in the analysis whenever appropriate. To reduce the variability due to different locations of lesions, patients with lesions underneath the electrodes (that could modify the induced electric field) will be excluded. Finally, as explained in the following, patients will only be included if they present with a working memory deficit, but no general intellectual impairment.

As the aim of this research is to improve working memory, only participants with a working memory impairment will be included. As we are focusing on improving a documented working memory deficit, we will not attempt to distinguish between pathologies (unless otherwise determined by the exclusion criteria for tDCS, e.g. a lesion underneath the electrodes), rather we will screen patients based on working memory performance. Final analysis will explore differences between pathologies. To identify a working memory impairment, cognitive tests from the Wechsler Adult Intelligence Test (WAIS-IV) and the Wechsler Memory Scale (WMS-IV) will be administered. These tests are administered to every patient as part of the routine care protocol. We will use the following scores:

- A WAIS-IV Full-Scale Intelligence Quotient (FSIQ) lower than 70: this score will identify any patients with significant intellectual impairment. The FSIQ will also be used as a covariate in the analysis to control for the effects of reduced overall intellectual ability rather than a specific working memory issue.
- A WAIS-IV Working Memory Index (WMI) score, obtained as a combination of the digit span score and the arithmetic score in the WAIS-IV, smaller than 85: this score identifies an auditory working memory impairment.
- A WMS-IV Visual Working Memory Index (VWMI) score, below 85 identifies weakness in visuospatial working memory.

The WAIS-IV and WMS-IV above are co-normed on a large sample (mea*n* = 100; SD = 15). To be included in the study, a patient will have a FSIQ > 70 and either a WMI or a VWMI or both < 85, which means we adopt a criterion score of one standard deviation below the mean. We use working memory indices that are a combination of auditory and visual working memory to identify an objectively defined working memory deficit. We will also run an exploratory post-hoc analysis factored by type of baseline WM deficit to ascertain if there are any modality specific benefits*.* Although this score reflects at least mild impairment, a weakness in working memory would be large enough to potentially improve with training.

### Sample size calculation

The chosen sample size is based partly on the available literature that examines the effects of cognitive training on healthy young and older volunteers and additional pilot data collected in our laboratory from healthy young and elderly adults. Specifically, a statistical power analysis was performed for sample size estimation, based on data from Au et al. [39], comparing the ACTIVE (right-tDCS, 20 subjects) to the CONTROL (SHAM, 22 subjects) group. The effect size (ES) in this study was 0.73, considered to be medium to large using Cohen’s criteria [55]. With an alpha = .05 and power = 0.80, the projected sample size needed with this effect size (GPower 3.1) is approximately *N* = 48 for this simplest between group comparison.

Evidence has shown that individuals with low initial performance respond better to brain stimulation [56]. As we expect ABI patients in our sample to have low baseline performance, due to our selection criteria, we may also expect them to respond better to the intervention, therefore showing larger effects. Assuming, as explained above, that larger effects could reasonably be expected in a clinical population, our proposed sample size of 40 will be adequate for the main objective of this study. A post hoc power analysis will be conducted to assess if the sample size is appropriate to detect differences in a clinical population such as ABI.

### Intervention

The BRAINSTORMING trial is designed as a randomized clinical trial, assessor and patient blinded with two parallel groups, and a simple randomization with a 1:1 allocation ratio. Participants identified as having reduced working memory will be randomly assigned to one of two experimental groups: an active (or right-sided tDCS) group and a control (or SHAM) group. Individuals included in the active group will receive 20 min of tDCS to the right dorsolateral prefrontal cortex while completing the WM training, whereas participants in the control group will complete the WM training while receiving sham (e.g., placebo) stimulation. Participant blindness to group membership will be achieved by the use of a SHAM protocol, which simulates current stimulation.

#### Randomization and blinding

A computer programme will generate forty random flags identifying the patient’s group (20 flags equal to 1 for ACTIVE, 20 flags equal to 0 for SHAM). Flag order will then be shuffled and a participant’s unique ID (sub-01, sub-02, … sub-40) associated to a flag, following the order of attendance. The study design is double blind: participants and the assessors will not know until the end of the intervention if the participant had received active or sham brain stimulation. A member of the research team (not the assessor) will program two stimulation protocols (active and sham) on the stimulation device, which will be set to operate into blind mode. As such, the person who administers the stimulation will be blind to the details of the protocol. Meaning they will not be able to see the details of the protocol. Patients are then randomly assigned to one or the other protocol, resulting in an intervention blind both to the assessor and the patient.

#### Brain stimulation protocol

**Fig. 1:**
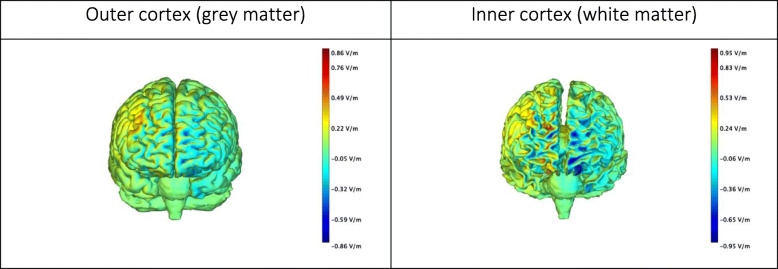
Frontal view of the simulated electric field (normal component (En) with tDCS configuration used in the study. The simulated electric field was obtained with StimViewer (Neuroelectrics®), using the realistic head model described in Miranda et al. [60]. Briefly, tissue boundaries were derived from MR images (scalp, skull, cerebrospinal fluid (CSF) – including ventricles, grey matter and white matter) and the Finite Element Method was used to calculate the electric potential in the head, with circular electrodes with a 3.14 cm^2^ area. Tissues were assumed to be uniform and isotropic and values for their electric conductivity were taken from the literature. A positive value for the component of the electric field normal to the cortical surface means the electric field normal component is pointing into the cortex, and such a field would be excitatory. On the other hand, an electric field pointing out of the cortex (negative normal component) would be inhibitory. Details of the simulation parameters are taken from https://www.neuroelectrics.com/wiki/index.php/Simulating_tCS_Electric_Fields_in_the_Brain

Brain stimulation will be administered using a Starstim 8 brain stimulation device (Neuroelectrics®). Standardized locations derived from electroencephalography will be used to place the electrodes over the right dorsolateral prefrontal cortex (DLPFC). We will use a bipolar setup, includes two circular Ag/AgCl electrodes (area = 3.14 cm^2^) filled with conductive gel and placed on F4 (anode) and Fp1 (cathode). Reference electrodes will be attached to the earlobe and impedances will be measured throughout the stimulation and kept below 20 kΩ. We will use a total current intensity of 2 mA for 20 min, preceded by 30 s ramping up and followed by 30 s ramping down (total stimulation time = 21 s). With these parameters and Ag/AgCl electrodes (area of 3.14 cm^2^) we obtain a current density of approximately 0.6 mA/cm^2, slightly higher than the one obtained with larger electrodes, but still well below the threshold for risk of tissue damage [57–59]. The simulated current distribution obtained with such setup is shown in Fig. 1. During sham stimulation, we will use the same setup as in the active condition but after ramping up, the current will be brought back to zero and the ramp up/down process repeated 30 s before the end of the 21 min time interval (total sham stimulation time = 21 min). We have recently completed data collection with the same protocol in 28 elderly and another study on stroke patients is undergoing: in these participants, brain stimulation has been well received.

#### Working memory training

The working memory training task is an internet-based visuo-spatial adaptive n-back training program. The same training paradigm has recently been successfully used with stroke patients [61]. Players must attend to a visual stream of spatial information that is presented sequentially and look for matches. Participants will see a grid with eight spaces, with one space filled in on each trial and they will press a button upon seeing a “match” in position with ‘n’ trials before. Within each session, players begin at a level of *n* = 1 and can progress up to *n* = 6 based on their scores in the previous block (i.e., this program is adaptive (closely following Jaeggi et al. [24]). Each session consists of 20 blocks of 20 + n trials each (~ 1 min per block), and progression to the next block is self-paced by the participant. This training task will last for ~ 20 min.

#### Participants’ timeline

**Fig. 2:**
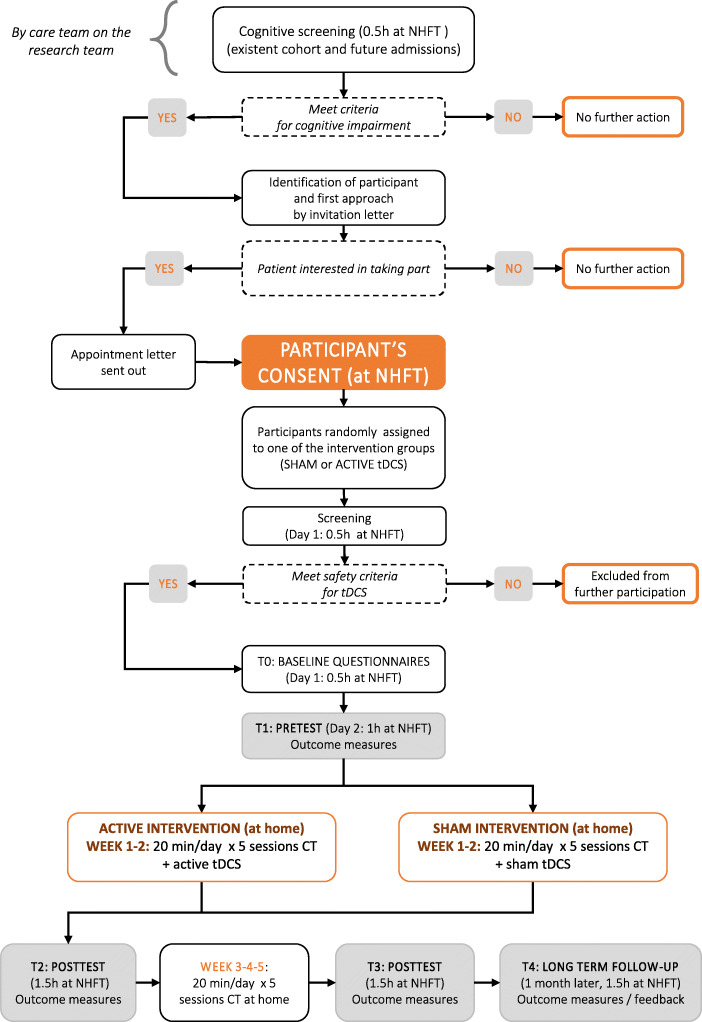
Protocol flowchart

The flowchart of the procedure is shown in Fig. 2, while a schedule of enrolment, interventions, and assessments is shown in Table 2. The care team as part of the research team identifies potential participants during the cognitive assessment, which is part of the routine care, and invites them to participate. Patients expressing an interest in the study are made aware of possible side effects of brain stimulation and asked to confirm their willingness to participate. During the first in-lab session (T0), following the informed consent procedure, each participant is randomly assigned to one of the two groups (active or sham stimulation) and interviewed by the assessor with a screening questionnaire, including questions about demographic information and health history to evaluate the inclusion/exclusion criteria. We also record information about current medications and information about time since injury, handedness and colour blindness. At this point, participants who don’t meet the eligibility criteria are excluded from further participation.

**Table 2:**
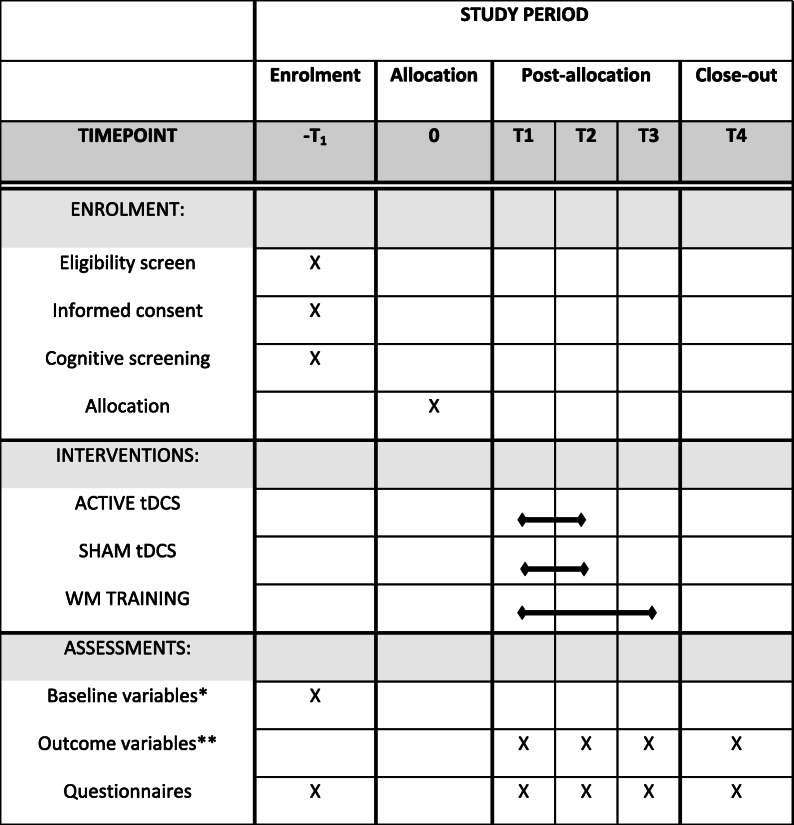
Schedule of enrolment, interventions, and assessments

*Digit span, Arithmetic span, symbol span, spatial addition, DalCAB

** Digit span, Arithmetic span, symbol span, spatial addition, DalCAB, training gains

##### Baseline session

After the informed consent process is carried out, eligible participants are administered the baseline measures (T0). The baseline data are included in data analyses as potential modifiers of performance. The baseline measures involve completing a series of questionnaires (Table 5). On the same day of baseline testing, patients are given a diary for them to keep. The diary contains information on how to setup and use the training program on their computer, education on how to do the working memory training, general troubleshooting information, and also a daily table to record information about the training session and any issue they might experience. The diary also contains contact details of the researcher administering the stimulation and information about future appointments. In addition, participants are asked to refrain from excessive alcohol or caffeine intake during the testing and to maintain good sleeping habits, where possible. In total, we expect the entirety of the first in-lab session (informed consent, screening, and baseline measures (T0)) to take about 1 h.

##### Outcome assessment (T1)

On the following day participants perform a series of pre-training outcome tasks (T1; Table 4), administered by an assessor, to measure their baseline working memory capacity and to assess, during and at the end of the training intervention, the efficacy of the training and the stimulation regime. These tests will not be used to determine eligibility. The assessor is blind to group assignment (control vs active).

On the same day as T1 testing, the assessor will familiarize them with the stimulation procedure (which is identical for sham or active) and the training game. The member of the team administering the training answers any questions about the study. Overall, this second session lasts for about 1 h.

##### Training phase 1

On the 10 consecutive training days after the T1 session, depending on the group, participants will receive active or sham brain stimulation (see section 3.5.2). As the assessor, who administer brain stimulation, is blind to the intervention, the procedure will be the same for both groups. At the same time, both groups complete the working memory training exercise for 20 min. If the WM training finishes before the stimulation, participants are asked to wait until the end of the tDCS session (20 min). Before each training session, participants are also asked to answer short questions (level of alertness, engagement, etc.). Once the training session and the stimulation are complete, the participant fills in a feedback form on the side effects of brain stimulation experienced, if any.

Participants will complete 10 consecutive training sessions (2 weeks, excluding weekends. Each session should take about 45 min (~ 10 min setting up of tDCS and ~ 20 min of WM training). This phase will be completed at home, with the assistance of the trainer. An attempt will be made to start the intervention on a Monday, therefore aiming to have breaks in the stimulation at the same point in the 2 weeks, and to keep the training time consistent over days. Nevertheless, administration time and date will be recorded. By the end of these 10 sessions, participants will be familiar with the training game and will be able to confidently undertake the training by themselves in the next 15 sessions.

##### Outcome assessment (T2)

When the first 10 days of training are complete, the participant will undergo time 2 (T2) assessment. As before, testing involves completing a series of computerised cognitive tasks to measure training gains and transfer (see 3.5.6). This assessment will also include the tests from the WAIS-IV and WMS-IV (Table 3).

**Table 3:** Screening tasks

Task	Description
Digit Span (WAIS-IV)	The patient is read a sequence of digits, to repeat forwards, backwards or in ascending order of magnitude (sequence).
Arithmetic (WAIS-IV)	The patient is read a number of mathematical problems increasing from very simple to complex in terms of the amount of information the person has to hold in mind.
Symbol Span (WMS-IV)	Patients are asked to look at a series of symbols for 5 s then choose which symbols they saw from a multiple-choice format, pointing to them in the order they were shown from left to right. The test progresses from only one symbol with the sequence increasing depending on how well people do.
Spatial Addition (WMS-IV)	The patient is shown a grid for 5 s, then a second grid for 5 s. The grids contain blue and red circles. The patient is provided with a number of cards showing red, blue or white circles and asked to place them onto a cardboard grid according to the following rules: Place a blue circle in any place where you saw a blue circle on only one of the pages. Place a white circle in any place where you saw a blue circle in the same place on both pages. Ignore red circles.

##### Training phase 2

Participants will then start the second training phase, involving 3 weeks of working memory training only (no brain stimulation). During this phase, patients will access the training program via internet at home on their own. Manualised weekly phone calls using a semi-structured script will be used to monitor the patients progress and to address any issue they might experience.

##### Outcome assessment (T3)

When the second 3-week training phase is complete, participants will undergo time 3 (T3) assessment, during which they repeat the same series of computerised cognitive tasks as in T2 (Table 4).

**Table 4:** List of outcome measures

TASK	DESCRIPTION	Target functions
Simple Reaction Time	Respond to each stimulus, with varying response-stimulus intervals.	Vigilance
Go/No-Go	It employs a continuous stream of two different stimuli for which a binary decision must be made, such that one stimulus type requires a response (go) and the other stimulus type requires the participant to withhold a response (no-go).	Executive control
2-Choice Reaction Time	Indicate the colour of each stimulus (2-choice responses; 50% each choice).	Vigilance
Dual task	Complete the 2-choice reaction time task while silently counting the number of each colour of stimuli presented. Count probe for one colour at the end of each set.	Executive control
Flanker	A central target stimulus is presented with flanking stimuli (flankers) on two sides that are either the same as (congruent) or different than (incongruent) the central target stimulus. The participant must decide and respond regarding a feature of the central stimulus (e.g., red or black) while ignoring/filtering the flanking stimuli.	Executive control
Item Working memory	Indicate whether a probe item was present or absent in a preceding study set of 2–6 items (50% present).	Working memory
Location working memory	Indicate whether a probe item was present or absent in the same position in a preceding study set of 2–6 locations (50% present).	Working memory
Visual search	Locate and indicate orientation (upright vs. inverted; 50% each) of a target among different shape distractors that are a different colour (feature search) or the same colour (conjunction search) as the target.	Orienting and Selection
Fixed visuo-spatial n-back task	Participants must attend to a visual stream of information that is presented sequentially and look for matches, pressing a button upon seeing a “match”. Three levels of difficulty will be used (n = 1,2,3)	Working memory

##### Outcome assessment (T4)

A final follow-up assessment, identical to the one at T2 and T3, will be carried out 1 month after the completion of the intervention, to assess maintenance of working memory improvements and transfer.

#### Cognitive tasks and questionnaires

A series of cognitive tasks will be administered at different time points and for a different purpose during the study: *screening*, *training*, and *outcome assessment*.

- Screening: the Digit Span, Arithmetic, Symbol Span, and Spatial Addition subtests from the WAIS-IV and WMS-IV will be used for screening participants for cognitive impairment (see 3.5.4, 35 min to complete). A description of the task is given in Table 3.
- Training Task: The visuo-spatial training task is a visuo-spatial adaptive n-back (see section 3.5.3).
- Outcome: 1) the Dalhousie Computerized Attention Battery (DalCAB, [68], 35 min to complete) will be used as the outcome assessment battery. A description of the tasks in the battery is provided in Table 4. For a detailed description of the parameters used in each task see [68]; 2) In addition, a non-adaptive visuo-spatial N-back task will also be used as outcome measure, with fixed levels of *N* = 1,2,3. (~ 30 min to complete); 3) The Digit span, Arithmetic span, Symbol span and spatial addition will also be used as outcome measures.
- A series of questionnaires and feedback forms will also be administered at single or multiple time points throughout the intervention to record variables that may impact or may be affected by performance on the task. Permission to use these instruments has been obtained for those questionnaires requiring it. A list of the questionnaires, together with a brief explanation and references is presented in Table 5.

**Table 5:** Questionnaires

	Questionnaire	Taken at
*Individual lifestyle*	Participant Health History (including details on injury, medications etc..)	T1
	Epworth Sleepiness Scale (to report habitual sleepiness [62]	
	Quality of life assessment (The Whoqol Group [63]), − in four facets (Physical health, Psychological, Social relationships, Environment)	
*Individual cognitive state*	Cognitive Failures Questionnaire (CFQ, [64]) to measure self-reported failures in perception, memory, and motor function.	T1
	Hospital Anxiety and Depression Scale (HADS) to measure emotional symptoms (HADS; [65])	
	Patient Reported Evaluation of Cognitive Status (PRECiS [66];)	T1/T3/T4
*Feedback on the of intervention*	Side effect of the tCS [57]	Every tCS session
	System usability scale (SUS [67];)	T3
	Attitude towards stimulation (to record how they feel their attitude towards the brain stimulation affected performance)	
	Feedback on strategy (to record whether they use a specific strategy to complete the training task)	
	Blinding questionnaire (to confirm efficacy of the blinding procedure)	T4

#### Outcome measures

Our primary outcome measure will be the training gain*,* obtained by looking at the relative change in n-level (# items maintained in working memory) over the course of the training, as well as performance increments in the pre−/post-test outcome measures in the stimulation relative to the control (SHAM) group. Transfer of improvement to other cognitive domains will be assessed by comparing performance in a battery of cognitive tasks given at different times points throughout and after the intervention. Maintenance of improvement will be assessed by comparing outcome measures (Table 4) at different occasions during and after the conclusion of the study.

- Training gain. Performance in training is measured as the N level reached on every session. The training gain is defined as the difference in N levels at two time points. Accuracies and reaction times at trial level will also be collected. In addition, we will administer a non-adaptive visuo-spatial task (N = 1, *N* = 2, *N* = 3), to allow us to quantify improvements at different testing points in terms of accuracy and reaction times in the trained cognitive domain.
- Transfer. We will use spatial tasks that test the same cognitive process as the training task to evaluate transfer to closely related tasks. We will use the same spatial addition and the symbol span task also used in the screening phase. We also will use the Dalhousie Computerized Attention Battery (DalCAB) [68], 8 cognitive tests to assess attentional functions in the vigilance, orienting and executive control attention network. Tasks are described in Table 4. Reaction time and accuracy will be computed for each task.
- Maintenance. To identify how long benefits of training and or stimulation are maintained in time, we will compare changes between outcome measures at T3 and at T4, 1 month after the end of the intervention.
- Everyday life improvement. It is important to understand whether improvement in cognitive tasks targeting specific processes can transfer to improvement in everyday life activities. We will use a self-reported questionnaire (PRECIS, Patchick et al., Clinical Rehabilitation, [66]) to evaluate if and how training in cognitive tasks targeting specific processes impacts on everyday activities.

### Data collection, management, analysis, and monitoring

Accuracies and reaction times at trial level will be collected, alongside the ‘n’ level for each block. Data will continuously be monitored for completeness and consistency. Data entry will be double-checked, and data quality will be ensured based examination of ranges. Prior to statistical analysis, we will test the distribution of the scores. We will analyse the data using appropriate statistical methods, including mixed and repeated measures ANOVAs, with factors of group and test variables. Variables potentially modulating the outcome of the intervention (such as age or time since injury) will considered as regressors in the analysis. Scores that are non-normally distributed will be analysed with non-parametric tests. Significant main effects and interactions (*p* < .05) will be followed by post-hoc tests corrected for multiple comparisons. Dependent variables on the computerized tasks will include mean reaction time (RT) and accuracy (% correct). The research team will analyse the data with a combination of SPSS, JASP, R, Python, and MATLAB.

### Risk assessment and monitoring

Common side effects in tDCS experiments are mild headache, itching, fatigue [69]. All experimenters using these techniques will undergo thorough training about how to deal with and minimize these risks. Clinical members of the research team will assess seriousness, causality (relatedness), an expectedness of any adverse event experienced by the participant with reference to the most recent regulatory tDCS guidelines [57]. Data on reported side effects will be reviewed following each treatment session by clinicians treating and supporting the participant during treatment. Following completion of intervention, course reported side effects will be reviewed by the PI and clinical co-PI. If none of the participants recruited to the study can tolerate the data collection or intervention, the trial will be stopped.

### Dissemination policy

Results of this study may be submitted for publication in a peer reviewed journal. On completion of the study, the data will be analysed and tabulated, and a final study report prepared and made available on ClinicalTrials.org, the study has been registered on the database. Results will be published in peer-reviewed journals and presented at relevant scientific meetings. Participants will be asked whether they would like to receive a copy of the final report, or a simplified version of it, and will be provided with this if they wish. Dissemination will be achieved through conference presentations, public talks and community talks. Upon publication, the anonymised dataset may be made available to other researchers upon request.

## Discussion

The overarching aim of the study is to assess if a specific form of non-invasive brain stimulation, namely direct current, has an impact on performance during a working memory training regimen in patients with an acquired brain injury. The novelty of our research resides in the combination of brain stimulation with working memory training: while the two interventions have been used before in clinical populations, to the best of our knowledge this is the first trial looking at the combination of both in acquired brain injury patients.

The focus of this study are chronic brain injury patients. These patients are no longer in a clinical setting but have returned home and are seen by the Community Brain Injury Service for community-based rehabilitation. These aspects make the proposed intervention particularly suitable for the population under study, as individuals can undertake the long (5 weeks) training regimen in the comfort of their home, at their own pace, minimizing the risk of drop-outs otherwise likely with long interventions. Our intervention is suitable for home healthcare, and, if proven successful, could represent a valuable asset to the NHS to address brain injury related cognitive impairment without putting excessive burden on patients and carers, and the NHS. At the same time, this trial will provide valuable knowledge to health authorities and the research community on the potential of non-invasive brain stimulation and training regimens in addressing chronic cognitive impairment in clinical populations. If successful, our study will provide evidence that a restorative approach to cognitive rehabilitation is possible, and an alternative to compensatory approaches currently used.

By evaluating performance at 1 month after the completion of the training regimen, we will shed new light on the long-term effects of cognitive training, also identifying potential modulatory effects of brain stimulation. This finding will, in turn, inform future protocols, in order to maximise improvement of cognitive functions while minimizing the amount of training necessary. Ultimately, patients would want these interventions to have an impact on their everyday life: we therefore also ask patients to self-evaluate (with the PRECIS questionnaire) how the training regimen impacted their cognitive functions and, indirectly, on their ability to cope with everyday situations.

Finally, our intervention falls within the remit of home healthcare medicine, with the potential to be fully self-administered by the patient (with support of carer if required) in their home. We will therefore collect data on the usability of the training, to inform future development in line with homecare technologies.

## Data Availability

Upon publication of the results, the datasets generated and/or analysed during the current study will be available from the corresponding author on reasonable request.

## Abbreviations

ABI: Acquired brain injury
tDCS: Transcranial direct current stimulation
ADHD: Attention deficit hyperactivity disorder
NHFT: Northamptonshire Healthcare Foundation NHS Trust
FSIQ: Full-Scale Intelligent Quotient
WAIS: Wechsler Adult Intelligence Scale
WMS: Wechsler Memory Scale
VWMI: Visual Working Memory Index
DalCAB: Dalhousie Computerized Attention Battery
DLPFC: Dorso-Lateral Pre-Frontal Cortex
CI: Chief Investigator
REC: Research Ethics Committee
PRECIS: Patient Reported Evaluation of Cognitive Status
HADS: Hospital Anxiety and Depression Scale
CFQ: Cognitive Failure Questionnaire
SUS: System Usability Scale
WHOQOL: World Health Organization quality of life assessment

## References

[1] Chiesa A, Calati R, Serretti A. Does mindfulness training improve cognitive abilities? A systematic review of neuropsychological findings. Clin Psychol Rev. 2011;31:449–64. 10.1016/j.cpr.2010.11.003 21183265

[2] Baddeley A (2012). Working memory: theories, models, and controversies. Psychology.

[3] Barbey AK, Koenigs M, Grafman J (2013). Dorsolateral prefrontal contributions to human working memory. Cortex.

[4] Smith EE, Jonides J, Koeppe RA (1996). Dissociating verbal and spatial working memory using PET. Cereb Cortex.

[5] Wager TD, Smith EE (2003). Neuroimaging studies of working memory: cognitive. Affect Behav Neurosci.

[6] Christodoulou C, DeLuca J, Ricker J, et al. Functional magnetic resonance imaging of working memory impairment after traumatic brain injury. J Neurol Neurosurg Psychiatry. 2001;71:161–8. 10.1136/jnnp.71.2.161 PMC1737512 11459886

[7] Nyberg L, Lövdén M, Riklund K, Lindenberger U, Bäckman L. Memory aging and brain maintenance. Trends Cogn Sci. 2012;16:292–305. 10.1016/j.tics.2012.04.005 22542563

[8] Rabinowitz AR, Levin HS (2014). Psychiatric clinics of North America. Psychiatr Clin North Am.

[9] Manktelow AE, Menon DK, Sahakian BJ, Stamatakis EA (2017). Working memory after traumatic brain injury: the neural basis of improved performance with methylphenidate. Front Behav Neurosci.

[10] Mattson AJ, Levin HS (1990). Frontal lobe dysfunction following closed head injury. A review of the literature. J Nerv Ment Dis.

[11] Dunning DL, Westgate B, Adlam A-LR. A meta-analysis of working memory impairments in survivors of moderate-to-severe traumatic brain injury. Neuropsychology. 2016;30:811–9. 10.1037/neu0000285 27182710

[12] Serino A, Ciaramelli E, Santantonio A, Malagù S, Servadei F, Làdavas E. Central executive system impairment in traumatic brain injury. Brain Inj. 2009;20:23–32. 10.1080/02699050500309627 16403697

[13] Lindeløv JK, Overgaard R, Overgaard M. Improving working memory performance in brain-injured patients using hypnotic suggestion. Brain. 2017. 10.1093/brain/awx001. 10.1093/brain/awx001 28335012

[14] McDowell S, Whyte J, D’Esposito M (1997). Working memory impairments in traumatic brain injury: evidence from a dual-task paradigm. Neuropsychologia.

[15] Smith CJ, Xiong G, Elkind JA, Putnam B, Cohen AS (2015). Brain injury impairs working memory and prefrontal circuit function. Front Neurol.

[16] Westerberg H, Jacobaeus H, Hirvikoski T, Clevberger P, Ostensson M-L, Bartfai A, Klingberg T. Computerized working memory training after stroke--a pilot study. Brain Inj. 2007;21:21–9. 10.1080/02699050601148726 17364516

[17] Sammer G, Reuter I, Hullmann K, Kaps M, Vaitl D (2006). Training of executive functions in Parkinson’s disease. J Neurol Sci.

[18] Klingberg T, Fernell E, Olesen PJ, Johnson M, Gustafsson P, Dahlström K, Gillberg CG, Forssberg H, Westerberg H (2005). Computerized training of working memory in children with ADHD-A randomized, controlled trial. J Am Acad Child Adolesc Psychiatry.

[19] Klingberg T (2010). Training and plasticity of working memory. Trends Cogn Sci.

[20] Noack H, Lövdén M, Schmiedek F (2014). On the validity and generality of transfer effects in cognitive training research. Psychol Res.

[21] van de Ven RM, Murre JM, Veltman DJ, Schmand BA (2016). Computer-based cognitive training for executive functions after stroke: a systematic review. Front Hum Neurosci.

[22] Toril P, Reales JM, Mayas J, Ballesteros S (2016). Video game training enhances visuospatial working memory and episodic memory in older adults. Front Hum Neurosci.

[23] Course-Choi J, Saville H, Derakshan N (2017). The effects of adaptive working memory training and mindfulness meditation training on processing efficiency and worry in high worriers. Behav Res Ther.

[24] Jaeggi SM, Buschkuehl M, Jonides J, Perrig WJ (2008). Improving fluid intelligence with training on working memory. Proc Natl Acad Sci.

[25] Åkerlund E, Esbjörnsson E, Sunnerhagen KS, Björkdahl A. Can computerized working memory training improve impaired working memory, cognition and psychological health? Brain Inj. 2013;27:1649–57. 10.3109/02699052.2013.830195 24087909

[26] Lundqvist A, Grundström K, Samuelsson K, Rönnberg J. Computerized training of working memory in a group of patients suffering from acquired brain injury. Brain Inj. 2010;24:1173–83. 10.3109/02699052.2010.498007 20715888

[27] Rolle CE, Anguera JA, Skinner SN, Voytek B, Gazzaley A. Enhancing spatial attention and working memory in younger and older adults. J Cogn Neurosci. 2017;29:1483–97. 10.1162/jocn_a_01159 PMC5903566 28654361

[28] Soveri A, Antfolk J, Karlsson L, Salo B, Laine M (2017). Working memory training revisited: a multi-level meta-analysis of n-back training studies. Psychon Bull Rev.

[29] Giordano J, Bikson M, Kappenman ES (2017). Mechanisms and effects of transcranial direct current stimulation. Dose-Response.

[30] Nitsche M, Paulus W (2000). Excitability changes induced in the human motor cortex by weak transcranial direct current stimulation. J Physiol.

[31] Paulus W (2003). Transcranial direct current stimulation (tDCS). Suppl Clin Neurophysiol.

[32] Thair H, Holloway AL, Newport R, Smith AD (2017). Transcranial direct current stimulation (tDCS): a beginner’s guide for design and implementation. Front Neurosci.

[33] Stagg CJ, Nitsche MA (2011). Physiological basis of transcranial direct current stimulation. Neuroscientist.

[34] Yavari F, Jamil A, Samani M, Vidor L, Nitsche MA (2018). Basic and functional effects of transcranial electrical stimulation (tES)—an introduction. Neurosci Biobehav Rev.

[35] Elmasry J, Loo C, Martin D (2015). A systematic review of transcranial electrical stimulation combined with cognitive training. Restor Neurol Neurosci.

[36] Passow S, Thurm F, Li S-C (2017). Activating developmental reserve capacity via cognitive training or non-invasive brain stimulation: potentials for promoting fronto-parietal and hippocampal-striatal network functions in old age. Front Aging Neurosci.

[37] Perceval G, Flöel A, Meinzer M (2016). Can transcranial direct current stimulation counteract age-associated functional impairment?. Neurosci Biobehav Rev.

[38] Andrews SC, Hoy KE, Enticott PG, Daskalakis ZJ, Fitzgerald PB (2011). Improving working memory: the effect of combining cognitive activity and anodal transcranial direct current stimulation to the left dorsolateral prefrontal cortex. Brain Stimul.

[39] Au J, Katz B, Buschkuehl M, Bunarjo K, Senger T, Zabel C, Jaeggi SM, Jonides J (2016). Enhancing working memory training with transcranial direct current stimulation. J Cogn Neurosci.

[40] Jeon S, Han S (2012). Improvement of the working memory and naming by Transcranial direct current stimulation. Ann Rehabil Med.

[41] Ruf S, Fallgatter AJ, Plewnia C (2017). Augmentation of working memory training by transcranial direct current stimulation (tDCS). Sci Rep.

[42] Mameli F, Fumagalli M, Ferrucci R, Priori A (2014). The stimulated brain. Part III: Improving functions in the atypical brain.

[43] Zimerman M, Hummel FC. Non-invasive brain stimulation: enhancing motor and cognitive functions in healthy old subjects. Front Aging Neurosci. 2010;2:149. 10.3389/fnagi.2010.00149 PMC2999819 21151809

[44] Nilsson J, Lebedev AV, Rydström A, Lövdén M. Direct-current stimulation does little to improve the outcome of working memory training in older adults. Psychol Sci. 2017;956797617698139. 10.1177/0956797617698139 PMC5536199 28509625

[45] Talsma LJ, Kroese HA, Slagter HA. Boosting cognition: effects of multiple session transcranial direct current stimulation on working memory. J Cogn Neurosci. 2016;29:1–14. 10.1162/jocn_a_01077 27897670

[46] Kang E, Kim D, Paik N (2012). Transcranial direct current stimulation of the left prefrontal cortex improves attention in patients with traumatic brain injury: a pilot study. J Rehabil Med.

[47] Jo J, Kim Y-H, Ko M-H, Ohn S, Joen B, Lee K (2009). Enhancing the working memory of stroke patients using tDCS. Am J Phys Med Rehabil.

[48] Villamar M, Portilla A, Fregni F, Zafonte R (2012). Noninvasive brain stimulation to modulate neuroplasticity in traumatic brain injury. Neuromodulation: Technolo Neural Interface.

[49] McLaren ME, Nissim NR, Woods AJ (2018). The effects of medication use in transcranial direct current stimulation: a brief review. Brain Stimul.

[50] Fujiyama H, Hyde J, Hinder MR, Kim S-J, McCormack GH, Vickers JC (2014). Delayed plastic responses to anodal tDCS in older adults. Front Aging Neurosci.

[51] Li LM, Uehara K, Hanakawa T (2015). The contribution of interindividual factors to variability of response in transcranial direct current stimulation studies. Front Cell Neurosci.

[52] Peterchev AV, Wagner TA, Miranda PC, Nitsche MA, Paulus W, Lisanby SH (2012). Fundamentals of transcranial electric and magnetic stimulation dose: definition, selection, and reporting practices. Brain Stimul.

[53] Brehmer Y, Li S-C, Müller V, von Oertzen T, Lindenberger U (2007). Memory plasticity across the life span: uncovering children’s latent potential. Dev Psychol.

[54] Karbach J, Könen T, Spengler M (2017). Who benefits the Most? Individual differences in the transfer of executive control training across the lifespan. J Cognitive Enhanc.

[55] Cohen J (1988). Statistical power analysis for the behavioral sciences.

[56] Tseng P, Hsu T-Y, Chang C-F, Tzeng OJL, Hung DL, Muggleton NG, Walsh V, Liang W-K, Cheng S-K, Juan C-H (2012). Unleashing potential: Transcranial direct current stimulation over the right posterior parietal cortex improves change detection in low-performing individuals. J Neurosci.

[57] Antal A, Alekseichuk I, Bikson M (2017). Low intensity transcranial electric stimulation: safety, ethical, legal regulatory and application guidelines. Clin Neurophysiol.

[58] Bikson M, Grossman P, Thomas C (2016). Safety of transcranial direct current stimulation: evidence based update 2016. Brain Stimul.

[59] Liebetanz D, Koch R, Mayenfels S, König F, Paulus W, Nitsche MA (2009). Safety limits of cathodal transcranial direct current stimulation in rats. Clin Neurophysiol.

[60] Miranda PC, Mekonnen A, Salvador R, Ruffini G (2013). The electric field in the cortex during transcranial current stimulation. NeuroImage.

[61] Ploughman M, Eskes GA, Kelly LP, Kirkland MC, Devasahayam AJ, Wallack EM (2019). Synergistic benefits of combined aerobic and cognitive training on fluid intelligence and the role of IGF-1 in chronic stroke. Neurorehab Neural Re.

[62] Johns MW (1990). A new method for measuring daytime sleepiness: the Epworth sleepiness scale. Sleep.

[63] The Whoqol Group (1998). The World Health Organization quality of life assessment (WHOQOL): development and general psychometric properties. Soc Sci Med.

[64] Broadbent D, Cooper P, FitzGerald P, Parkes K (1982). The cognitive failures questionnaire (CFQ) and its correlates. Br J Clin Psychol.

[65] Zigmond AS, Snaith RP (1983). The Hospital Anxiety and Depression Scale. Acta Psychiatrica Scandinavica.

[66] Patchick E, Vail A, Wood A, Bowen A (2016). PRECiS (patient reported evaluation of cognitive state): psychometric evaluation of a new patient reported outcome measure of the impact of stroke. Clin Rehabil.

[67] Brooke J, Jordan PW, Thomas B, Weerdmeester BA, McClelland AL (1996). SUS: a “quick and dirty” usability scale. Usability evaluation in industry.

[68] Jones SA, Butler BC, Kintzel F, Johnson A, Klein RM, Eskes GA (2016). Measuring the performance of attention networks with the Dalhousie computerized attention battery (DalCAB): methodology and reliability in healthy adults. Front Psychol.

[69] Poreisz C, Boros K, Antal A, Paulus W. Safety aspects of transcranial direct current stimulation concerning healthy subjects and patients. Brain Res Bull. 2007;72:208–14. 10.1016/j.brainresbull.2007.01.004 17452283

