# Publisher Correction to: BRAINSTORMING: A study protocol for a randomised double-blind clinical trial to assess the impact of concurrent brain stimulation (tDCS) and working memory training on cognitive performance in Acquired Brain Injury (ABI)

**DOI:** 10.1186/s40359-021-00516-7

**Published:** 2021-01-28

**Authors:** Sara Assecondi, Rong Hu, Gail Eskes, Michelle Read, Chris Griffiths, Kim Shapiro

**Affiliations:** 1 Visual Experience Laboratory, School of Psychology, University of Birmingham, Birmingham, UK; 2 Center for Human Brian Health (CHBH), University of Birmingham, Birmingham, UK; 3 Department of Neurology, Guangzhou First People’s Hospital, School of Medicine, South China University of Technology, Guangzhou, Guangdong China; 4 Departments of Psychiatry and Psychology & Neuroscience, Dalhousie University, Halifax, NS Canada; 5 Northamptonshire Healthcare NHS Foundation Trust, Northampton, UK

## Publisher Correction to: BMC Psychology (2020) 8:125 10.1186/s40359-020-00454-w

Following publication of the original article [1], the authors flagged that the article had published with the Acknowledgements erroneously excluded from the declarations at the end of the article.

Please be advised that the Acknowledgements are as follows:

“We would like to thank Jacob Kroeker, Katie McKearney, Arthur MacDonald and funding support from the Atlantic Canada Opportunities Agency (Atlantic Innovation Fund) for the development of the working memory training protocol. RH is supported by a Guangzhou Planned Project of Science and Technology grant (2016201604030018).”

The original article has now been corrected to include this declaration.

The publisher apologizes for any inconvenience caused.
